# Comparison of follicular fluid and serum levels of Inhibin A and Inhibin B with calculated indices used as predictive markers of ovarian hyperstimulation syndrome in IVF patients

**DOI:** 10.1186/1477-7827-7-86

**Published:** 2009-08-24

**Authors:** Jiri Moos, Karel Rezabek, Vanda Filova, Martina Moosova, Jana Pavelkova, Jana Peknicova

**Affiliations:** 1 Assisted Reproduction Centre, Department of Obstetrics and Gynecology, General Teaching Hospital in Prague, Czech Republic; 2 Sigma-Aldrich spol. s r.o., Prague, Czech Republic; 3 Immunotech a.s., Prague, Czech Republic; 4 Laboratory of Diagnostics for Reproductive Medicine, Institute of Biotechnology, Academy of Sciences of the Czech Republic v. v. i., Prague, Czech Republic

## Abstract

**Background:**

Ovarian Hyperstimulation Syndrome (OHSS) is a severe health complication observed in some patients undergoing hormonal stimulation during IVF. Presence of OHSS is often associated with a high count of growing follicles responding to FSH hyperstimulation. However, the number of responding follicles may not be sufficient enough to predict the onset and severity of OHSS. The aim of this study was to find whether follicular fluid (FF) and serum concentrations of Inhibin A and Inhibin B in patients undergoing IVF treatment may serve as a predictor of OHSS status independent of the growing follicles count.

**Methods:**

Serum and follicular fluid of fifty-three women undertaking the IVF program were separated into four groups according to their OHSS status and growing follicles count and analyzed for serum and FF concentrations of Inhibin A and Inhibin B. The resulting data were combined with clinical and demographic data to calculate indices independent of the growing follicles count.

**Results:**

Serum Inhibin A and Inhibin B concentrations showed no significant difference between the severe OHSS group and the control group without OHSS. Moreover, the serum concentrations of Inhibin A and Inhibin B were strongly correlated with the growing follicles count. Their concentrations in the high responders group (>18 follicles) were significantly higher (p < 0.00001, p < 0.0001) when compared with normal and low responders (<18 follicles). To suppress the dependence on the growing follicle count, three indices were constructed and calculated. The best association with OHSS status and independence of the growing follicle count was achieved by using the Inhibin B TFF/SBM index calculated as follows: [concentration in FF] × [growing follicle count]/[concentration in serum] × [body mass]. The Inhibin B TFF/SBM index showed a clear difference (p = 0,00433) between the group with severe OHSS and the control group, while showing no apparent correlation with the growing follicle count.

**Conclusion:**

These observations demonstrated that while neither serum nor FF concentrations of Inhibin A nor Inhibin B can be used as an OHSS predictor independent of the growing follicle count, calculated indices may meet the criteria.

## Background

Ovarian Hyperstimulation Syndrome (OHSS) is a severe health complication observed in the luteal phase of some patients undergoing hormonal stimulation during the IVF cycle [1]. The syndrome is characterized by an increase in size of the ovaries associated with a dramatic increase in vascular permeability causing ascites and eventually more severe complications [2,3]. The severe form of OHSS, characterized by haemoconcentration, thrombosis, oliguria, dysponea and tachycardia [3,4] requires patient hospitalization and constitutes a potentially lethal complication of infertility treatment for otherwise healthy women [5].

Embryo transfer and the resulting pregnancy have a negative impact on the severity of OHSS, so in the case of increased risk of the development of the severe or even critical form of OHSS, the embryo transfer is often cancelled and embryos have to be cryo-preserved. However, cryopreservation of all embryos is not a preferred method by infertility physicians [6]. An accurate and reliable OHSS prediction tool is thus needed.

However, our knowledge of OHSS pathogenesis is far from complete and predictive factors of OHSS are not efficient enough. The only common denominator in OHSS pathology is the role of hormonal hyperstimulation by FSH or Clomifene citrate followed by hCG administration (a common substitute of natural LH in IVF cycles), which is considered a triggering factor of OHSS. In FSH-stimulated ovaries, a peak of hCG may cause a hypersensitive response resulting in complications as described above [7]. The potential OHSS biomarkers should be thus expected among proteins and small molecules synthesized and/or modified in the ovary in response to FSH and hCG administration.

Several candidates have been proposed as potential biomarkers of OHSS, e.g. VEGF and VEGF receptor [8], Inhibin [9], AMH [10,11]. However, some of these potential biomarkers (Inhibin B, AMH) may predict OHSS because they predict the number of responding follicles. Enskog et al [9] took into account the relation between potential biomarkers and matched the OHSS patients and the controls in age and follicle count. The number of responding follicles showed poor predictive value in several other studies [2,3].

To test a potential association with OHSS status, several proteins involved in FSH signaling in ovarian follicles (PAPP-A, IGFBP4, IGF-1, Inhibin A, Inhibin B) have been tested in preliminary experiments. Based on the results of preliminary experiments, two proteins secreted by ovarian granulosa cells in response to FSH stimulation - Inhibin A and Inhibin B [12] have been chosen.

Inhibin secretion by granulosa cells and the evidence that ovarian Inhibin A and Inhibin B suppress FSH production has been reported by Ericsson and Hsueh [13]. Inhibins are heterodimeric glycoprotein hormones composed of one α (18 kDa) and one β (14 kDa) chain linked by disulphide bonds. Inhibin A consists of α-β_α _subunits and Inhibin B consists of α-β_β _subunits [14]. Meunier et al. [15] reported the expression of Inhibin subunits in various tissues, the Inhibin α subunit, however, is predominantly expressed in the gonads.

The aim of our present study was to find whether FF and serum concentrations of Inhibin A and Inhibin B in patients undergoing IVF treatment are correlated with the presence or absence of OHSS and how are those parameters related to the number of responding ovarian follicles. FF and serum concentrations of Inhibin A and Inhibin B were analyzed in IVF patients with a high ovarian response (>18 follicles) with respect to the OHSS status. Samples were collected at the time of the oocyte retrieval. Concentrations of Inhibin A and Inhibin B in paired FF and serum samples were also used in combination with clinical and demographic data to calculate indices which were evaluated as independent parameters.

## Methods

### Female patients

Patients undergoing regular stimulation for IVF were recruited for the study at the Centre of Assisted Reproduction, Dept. of Obstetrics and Gynecology, General Teaching Hospital in Prague. A total of 376 women were recruited for the study between March 2006 and October 2007. Samples of patients who did not agree with blood drawing or failed to deliver a good quality serum and/or follicular fluid sample were not selected for Inhibin A and Inhibin B analysis.

Patients were separated into four groups based on the number of growing follicles and OHSS status. Groups designated OHSS-2 and OHSS-3 included patients with a high number of growing follicles (the number of growing follicles ranged from 18 to 57 follicles). Patients in group OHSS-2 suffered from moderate OHSS and patients in group OHSS 3 showed symptoms of severe OHSS. No patient suffering from a critical form of OHSS was observed during the study, so no such sample is included.

Patients for the control OHSS-1 (>18 follicles) group were selected to match the parameters of OHSS 3 group (Age 24-34 years old, average number of growing follicles = 29.1, mean BMI = 22.3) as closely as possible. Patients in the other control group (OHSS-1, <18 follicles) included patients with a growing follicle count ranging from 6 to 18. Those patients were also selected to match the parameters (age and BMI) of OHSS 3 group.

The relation between the number of responding follicles in individual patients and the OHSS status was evaluated (without any patient intervention and Inhibin analysis) in a cohort of 567 consecutive patients collected in 2007 (January to December).

Follicular fluid and serum samples were obtained with the patient's permission. All the patients participating in this study signed an Institutional Board approved Informed Consent form.

All subjects underwent a standard treatment protocol - FSH ovarian hyperstimulation using GnRH long agonists or GnRH short antagonist's protocol with hCG induction of the follicular/egg maturation 36 hours before the collection of the egg. Optimal FSH dose was chosen individually according to the antral follicle count and growing follicle monitoring.

### Growing follicle count

The number and size of follicles responding to FSH stimulation was regularly monitored by transvaginal sonography measurements. The growing follicle count used in this study was obtained at the day of oocyte pickup and includes all follicles > 12 mm.

### OHSS classification

Patients were classified by using the criteria of Navot et al [2] and separated into 3 groups. For the purpose of this study, patients with mild OHSS were not analyzed separately and the control groups (OHSS 1) contained both patients without symptoms of OHSS as well as patients with mild OHSS. The moderate OHSS group (OHSS 2) was characterized by abdominal distention and discomfort, nausea, ascites and ovarian size > 5 cm (determined by sonography). Patients with severe OHSS (OHSS 3 group) were characterized by massive ascites, haemoconcentration (hematocrit >45%, WBC > 15000/μl), oliguria, creatinine >130 μmol/l, creatinine clearance < 50 ml/min and enlarged ovaries.

### Follicular fluid aspiration and blood processing

FF was obtained from the puncture of ovarian follicles (14 to 22 mm in diameter). After the oocytes were removed, the FF was pooled, cleared by centrifugation, and the resulting supernatant was transferred into sterile tubes, frozen at -20°C and stored at -70°C for further analysis. Samples of FF visibly contaminated with blood were excluded from the study. In parallel, samples of blood (5 ml) were taken on the day of oocyte retrieval, allowed to clot, cleared by centrifugation, and the resulting sera were frozen at -20°C and kept at -70°C until assayed.

### Immunoassays

Serum and FF concentrations of Inhibin A and Inhibin B were analyzed using ELISA kits (DSL, A Beckman Coulter Company, Webster, TX, USA). The immunodiagnostic kits were processed according to the manufacturer's instructions. For the analysis of FF Inhibin A and Inhibin B, the FF samples were diluted 200-fold with calf plasma (Scantibodies Laboratory, Inc., Santee, CA, USA). The linearity of the dilution was verified (recovery percentages obtained within the range of 84.6% to 109%).

### Statistical analysis

The concentration data and the calculated indices in each group were expressed as arithmetic mean +/- SD. The resulting data were tested for statistical significance using the Mann-Whitney U test and the statistical significance was set at p < 0,05. Data were processed using the NCSS 2007 software (Number Cruncher Statistical Systems, Kayswille, UT, USA).

## Results

### OHSS and growing follicle count

The relation between the number of responding follicles in individual patients and the OHSS status was evaluated in a cohort of 567 consecutive patients collected in 2007 (Table 1). The patients were separated into three groups based on the growing follicle count. The low responders' group was characterized by less than 6 responding follicles, in the group of normal responders, the growing follicle count ranged from 6 to 18 follicles and patients with a growing follicle count above 18 were considered high responders. Among the total of 567 patients, 13 patients with severe OHSS (OHSS 3 group) have been found, and the majority (11) of them was present within the group of high responders. Within the group of normal responders, OHSS 3 patients constitute less than 1% of patients.

**Table 1:** Distribution of 567 consecutive patients into low, normal and high responder groups

	**Low responders (<6 follicles) (%)**	**Normal responders (6-18 follicles) (%)**	**High responders (>18 follicles) (%)**	**Total (%)**
**n - Total**	137 (100)	305 (100)	125 (100)	567 (100)
**n - OHSS 1**	137 (100)	299 (98)	103 (83.4)	539 (95.1)
**n - OHSS 2**	0	4 (1.3)	11 (8.8)	15 (2.6)
**n - OHSS 3**	0	2 (0.7)	11 (8.8)	13 (2.3)

n = number of patients

The count and percentages of patients based on OHSS status were calculated in each responder group independently

### Inhibin A and Inhibin B levels

The presented data clearly demonstrate that in a group of high responders, the growing follicle count is only a weak indicator of OHSS status. To obtain an additional predictive marker of OHSS independent of the growing follicle count, paired samples of follicular fluid (FF) and blood serum (drawn on the day of oocyte retrieval) were analyzed for Inhibin A and Inhibin B concentrations. In the first experiment, only the data of patients with no or mild OHSS status (OHSS 1) were analyzed. The group of normal responders (6-18 growing follicles) was compared with the data of high responders - Table 2.

**Table 2:** Serum and FF concentrations and calculated indices in high responders (>18 follicles) and normal and low responder groups (<18 follicles)

	**OHSS 1, >18 follicles** **(n = 18)**	**OHSS 1, <18 follicles** **(n = 18)**
**Number of Follicles**	31.6 ± 9,72	10.6 ± 2,76* ^6^
**Inhibin A FF **[pg/mL]	52536 ± 15184	42350 ± 13137
**Inhibin A Serum **[pg/mL]	405 ± 125	155 ± 98.5 * ^5^
**Inhibin B FF **[pg/mL]	60960 ± 24105	45923 ± 15689
**Inhibin B Serum **[pg/mL]	288 ± 141	109 ± 66.3 * ^4^
Calculated Indices		
**Inh A OFC**	13.7 ± 5.87	14.9 ± 7.90
**Inhibin A OFC-BM**	884 ± 334	947 ± 500
**Inhibin A TFF/SBM**	64.9 ± 24.8	49.8 ± 16.2
**Inh B OFC**	9.45 ± 4.56	11.5 ± 9.79
**Inhibin B OFC-BM**	619 ± 302	729 ± 601
**Inhibin B TFF/SBM**	111 ± 48.5	121 ± 119

Presented data are arithmetic means ± standard deviations

Significance: *4 *p *< 0.0001; *5 *p *< 0.00001; *6 *p *< 0.000001

The serum concentrations of both Inhibin A and Inhibin B were significantly different in both groups (p < 0.00001 for Inhibin A and p < 0.0001 for Inhibin B). That observation clearly demonstrates a dependence of serum Inhibin A and Inhibin B concentrations on the growing follicle count.

### Calculated indices

To compensate for the effect of a growing follicle count and to obtain independent parameters, three indices were constructed and calculated. One Follicle Contribution (OFC) index simply divides the serum concentration with the growing follicle count.



OFC-BM index is calculated in a similar way, but also considers the patient body mass.



A different philosophy was used to calculate the TFF/SBM index. The index reflects a distribution of total inhibin between FF and blood and is calculated as follows:



The constructed indices have shown no significant difference between the high responder and normal responder groups, so the same analysis was performed as a retrospective study in the groups suffering a moderate (OHSS 2) and severe (OHSS 3) form of OHSS - Table 3.

**Table 3:** Serum and FF concentrations and calculated indices in OHSS 1, OHSS 2 and OHSS 3 groups

	OHSS 3, >18 follicles (n = 10)	OHSS 2, >18 follicles (n = 7)	OHSS 1, >18 follicles (n = 18)
Number of Follicles	29.1 ± 5.17^a4^	37.8 ± 10.2	31.6 ± 9.72
**Inhibin A FF **[pg/mL]	49236 ± 14388	49172 ± 15688	52536 ± 15184
**Inhibin A Serum **[pg/mL]	553 ± 222	456 ± 155	405 ± 125
**Inhibin B FF **[pg/mL]	46082 ± 19025	55383 ± 25435	60960 ± 24105
**Inhibin B Serum **[pg/mL]	373 ± 185	391 ± 199	288 ± 141

Calculated Indices			

Inhibin A OFC	18.9 ± 7.29	12.8 ± 5.95	13.7 ± 5.87
Inhibin A OFC-BM	1158 ± 346*,^a^	749 ± 332	884 ± 334
Inhibin A TFF/SBM	44.2 ± 12.2 *,^a^	71.8 ± 24.7	64.9 ± 24.8
Inhibin B OFC	12.70 ± 5.66	10.6 ± 5.78	9.45 ± 4.56
Inhibin B OFC-BM	807 ± 395	616 ± 315	619 ± 302
Inhibin B TFF/SBM	62.0 ± 23.8* ^2, a^	96.9 ± 35.3	111 ± 48.5

Presented data are arithmetic means ± standard deviations.

Significance between OHSS1 and OHSS 3 groups: *p < 0.05; * ^2^p < 0.01.

Significance between OHSS 1 and OHSS 2 groups: ^a^p < 0.05, ^a4^p < 0.0001.

Both the Inhibin A OFC-BM and TFF/SBM indices can distinguish the OHSS 3 group from the control group and also from the OHSS 2 group at the p < 0.05 level. Better significance was achieved by using the Inhibin B TFF/SBM index. Inhibin B TFF/SBM index can distinguish the OHSS 3 group from the control group at p < 0.01 and the OHSS 3 from the OHSS 2 group at p < 0.05.

Although the Inhibin A OFC-BM and TFF/SBM indices may be also useful as severe OHSS indicators, the Inhibin B TFF/SBM index is particularly promising as an OHSS indicator independent of the growing follicle count. Inhibin B TFF/SBM index combines a highly significant difference between the severe OHSS group and the control group with no apparent correlation with the growing follicle count.

## Discussion

In the present retrospective study, FF and serum concentrations of Inhibin A and Inhibin B were analyzed with respect to OHSS status in patients undergoing regular IVF treatment. In contrast to most studies where the basal serum levels of Inhibin B on Day 3 to Day 5 are correlated with the expected ovarian response and IVF outcome [16-20], our present study is one of the few analyzing levels of Inhibin A and Inhibin B at the time of oocyte pickup. Both FF and serum concentrations in paired samples are required to calculate the TFF/SBM index and the paired FF and serum samples can only be obtained at the day of oocyte retrieval. The use of paired samples is a useful tool to study ovarian hormones. For instance, it has been demonstrated that the amount of FF Inhibin A is correlated with its serum concentration in paired samples [21], an observation confirming the hypothesis that the ovary is a principal source of inhibin A in serum of non-pregnant women [22].

Some of the potential biomarkers of OHSS, namely Inhibin B and Anti Muellerian Hormone (AMH), can predict the OHSS status because they are good indicators of ovarian response to FSH stimulation and can thus predict the number of responding follicles [16,23,24]. The growing follicle count is, in turn, correlated with the OHSS status, since the high number of growing follicles is a necessary prerequisite to OHSS development [25,26].

Enskog et al [9] has monitored the serum levels of Inhibin A and Inhibin B from the beginning of ovarian stimulation until 3 days post embryo transfer. They observed a higher Inhibin B concentration in the OHSS group during the gonadotropin stimulation and also at the day of oocyte retrieval. Inhibin A was found to be elevated only after the OHSS onset. Our present study has only partially confirmed those observations. We have found that both serum Inhibin A and Inhibin B was elevated in the OHSS group at the day of oocyte retrieval, but that only Inhibin A was elevated significantly. Moreover, Inhibin A serum concentration depends on the FF volume [21,27] which is a function of a growing follicle count. Babayof et al [28] was studying the difference between hCG and GnRH agonists in triggering the oocyte maturation in IVF patients with polycystic ovaries. They also reported that elevated serum Inhibin A levels during the luteal phase are associated with a higher risk of OHSS in the hCG group. However, only four patients with OHSS were reported in the hCG group, so the possible association of severe OHSS with elevated serum Inhibin A remains uncertain.

Considering these observations and our own results, we have found that the serum concentration of inhibins is not a very reliable predictor of OHSS. FF concentrations of both Inhibin A and Inhibin B were in agreement with previous reports [29,30], but did not show any clear association with OHSS status.

Due to the observations described above, we decided to combine FF and serum concentrations and other clinical data to eliminate the effect of a variable number of growing follicles and to obtain an independent OHSS predictor.

To calculate the contribution of a single follicle to the serum inhibin concentration (OFC index) was an obvious strategy, but brought only moderate improvement over the serum concentration data. When the patient's body weight was also considered and used to calculate the OFC-BM index, the effect of body mass was only moderate. This observation is not very surprising, since controversial reports showing the correlation of body weight and/or BMI with OHSS status are available [3,31,32].

The best result has been obtained by using TFF/SBM index, particularly with the Inhibin B TFF/SBM index.

TFF/SBM index reflects the ratio between the total amount of FF inhibin and serum inhibin concentration multiplied by body mass. The total amount of FF inhibin was estimated by using the growing follicle count. The more accurate result would probably be obtained by using the total FF volume instead of the growing follicle count [21], but from a practical point of view, obtaining the growing follicle count appears to be more convenient in a daily laboratory routine than the total FF volume measurement. Moreover, the use of the growing follicle count provides sufficient results.

The differences in OHSS predictive value between Inhibin A and Inhibin B were probably due to the fact that their serum concentrations display a different time course during the menstrual cycle [33]. While Inhibin B increases gradually following the FSH stimulation of the granulosa cells and is already declining at the time of oocyte retrieval, Inhibin A is only elevated at the time of the LH surge and stays high even after the time of oocyte pickup. In this respect, our observations are in accordance with those reported by Enskog et al [9].

During the 20 months of recruitment, 10 complete FF and serum samples of OHSS 3 patients and 7 samples of OHSS 2 patients were collected. The number of OHSS 3 and OHSS 2 samples is too low to perform a more detailed statistical analysis and we are not able to collect a sufficient amount of samples in a reasonable timeframe. The result of this study should be thus considered as a preliminary work. A larger, preferably multicentric study is needed to confirm our observations.

## Conclusion

The observations presented in this study demonstrate that both serum and FF concentrations of Inhibin A and Inhibin B have no or only weak association with OHSS status. Moreover, Inhibin A and Inhibin B serum concentrations are strongly correlated with the growing follicle count and thus cannot be considered as a reliable OHSS biomarker. All the calculated indices appear to be better indicators of OHSS status than serum and FF inhibin concentrations. Inhibin B TFF/SBM index is a particularly promising candidate. It combines the highly significant difference between the severe OHSS group and the control group, while showing no apparent correlation with the growing follicle count. Due to the low number of OHSS 3 and OHSS 2 (10 and 7 respectively) samples collected during the 20 months of recruitment, the data should be considered as preliminary.

## Competing interests

VF is and JM was (until Dec 2007) an employee of Immunotech a.s., a subsidiary of Beckman Coulter Inc. Beckman Coulter is a company which manufactures and holds patent rights to Inhibin A and Inhibin B diagnostic kits.

Immunotech a.s. also provided partial support for the present study.

The remaining authors declare that they have no competing interests.

## Authors' contributions

JM designed and coordinated the study and was responsible for data analysis and interpretation, and for manuscript preparation. KR was responsible for clinical data acquisition, management and supervision of the manuscript presentation. VF carried out the immunochemical testing of Inhibin A and Inhibin B levels and was also responsible for sample management. JPa and MM were responsible for sample acquisition and preparation, and participated on data acquisition and interpretation.

JPe participated on the study design and helped to draft the manuscript. All authors read and approved the final manuscript.
